# Living to Work (from Home): Overwork, Remote Work, and Gendered Dual Devotion to Work and Family

**DOI:** 10.1177/07308884231207772

**Published:** 2023-10-23

**Authors:** Kim de Laat

**Affiliations:** 1 Stratford School of Interaction Design and Business, 8430 University of Waterloo, Stratford, ON, Canada

**Keywords:** devotion schemas, dual devotion, work-family conflict, flexible work arrangements, remote work, gender inequality, overwork

## Abstract

Contemporary North American work culture is characterized by experts as one of overwork. Throughout much of the previous century, many parents devoted themselves either to their careers, or to their families. These “competing devotions” served as a cultural model for making sense of the world and alleviated the tension between overwork and family life. Data from interviews with 84 IT workers are used to examine whether devotion to work and family is still experienced as oppositional for working parents. I find that interviewees report feeling devoted both to their families and their careers, which I refer to as dual devotion. Such espousals of dual devotion are facilitated by the use of flexible work policies—remote work and flextime—which enable those with dual devotions to accomplish work–life integration. However, whereas men perceive remote work as allowing them to dedicate more time to childcare, women perceive it as allowing them to dedicate more time to work. These findings advance our understanding of the relationship between gender inequality and the experiential dimensions of work and family time: the practices that enable dual devotions, in particular remote work, help parents maintain an orientation to time that makes overwork more palatable. In either case, workplaces win since women are working long hours and men are not sacrificing paid work hours to take on more childcare or housework.

In the twenty-first century, people laboring in a variety of industries work harder and longer than one might reasonably expect, and this often happens with their consent (Golden, 2006, 2009). Examining the factors enabling women's support for long work hours, Mary Blair-Loy (2003) identified the role of devotion schemas: devotion to work and devotion to family. Work devotion entails intense time commitments and a strong emotional allegiance to one's employer. Its avatar is the “ideal worker’ who prioritizes paid work above all else and fulfills workplace demands regardless of the time required (Acker, 1990; Davies & Frink, 2014; Williams, 2010). Family devotion also demands hard work, albeit work that is undervalued and unpaid (Folbre, 1995, 2006). It is gendered, through the assignment of responsibility for housework and childcare to women.

Historically, work and family devotion have been seen as conflicting forces; being devoted to one sphere generally means being less devoted to the other. However, shifts in labor market demographics call into question the extent to which these cultural models are experienced as competing (Dow, 2016; Moen et al., 2013). While working part-time was once a common strategy for women who adhere to family devotion but need to earn income, more women are working full-time (Collins, 2019; Damaske, 2011). Ideas about what it means to be a ‘good father’ have also shifted, and there are cultural and political efforts to promote fathers’ involvement in childcare (Ball & Daly, 2012; Raley et al., 2012). Although mothers continue to take on the bulk of the second shift, there is a growing convergence between the amount of time that mothers and fathers spend with children during the week (Guppy et al., 2019; Kaufman, 2013). Such shifts occur within a cultural context characterized by overwork, the practice of working 50 h or more per week (Cha & Weeden, 2014).^1^ Overwork is particularly common in professional and managerial occupations, where long working hours are deeply ingrained in organizational cultures and routines (Cha & Weeden, 2014; Kelly & Moen, 2020; Padavic et al., 2020). This suite of changes motivates the present study to examine why, in the current era of overwork and the increasing demands of parenthood, workers persist in working as long as they do, and the role devotion schemas play in this process.

Through interviews with 84 IT employees in a large financial services firm, I found that many working parent interviewees espouse what I refer to as* dual devotion*, a strong sense of devotion both to their families and to their careers. Flexible work arrangements, particularly remote work, facilitate dual devotion by enabling individuals to integrate their work and family lives. However, men and women perceive remote work differently: men see it as an opportunity to dedicate more time to childcare, while women view it as a means to allocate more time to work. In both cases, employers win since neither men nor women are sacrificing overwork as they remain highly involved in childcare and housework. These findings advance our understanding of the relationship between gender inequality and the experiential dimensions of work and family time; the practices that enable dual devotions, in particular remote work, help parents maintain an orientation to time that makes overwork more palatable.

## Overwork and Devotion Schemas

Contemporary North American work culture is characterized by experts as one where overwork is the norm (Blair-Loy & Cech, 2017; Jacobs & Gerson, 2004; Kelly & Moen, 2020; Padavic et al., 2020; Schor, 1998). In the United States, where overworking is particularly common, the annual average number of hours worked has increased by 183 h since 1975, equivalent to one month of extra work (Mishel, 2013). Overwork spans the labor market, from gig workers balancing multiple jobs and working 10-h days to make ends meet, to medical staff working 24-h shifts, to technology workers with 24/7 availability (Berdahl et al., 2018; Perlow, 2012; Ravenelle, 2019). Many are expected to do more with fewer resources, in unfavorable and unpredictable working conditions, often with no benefits (Gerstel & Clawson, 2018; Jacobs & Gerson, 2004; Vosko, 2011).

While overwork is imposed from above by greedy institutions (Coser, 1974), it may also be self-imposed; among a certain class of workers who love what they do, many self-select into working long hours and accept it as normal (Moen et al., 2013). Blair-Loy (2003) refers to this as work devotion: a cultural model used for making sense of the world, in which work is viewed as a vocation that requires single-minded focus, and from which adherents derive meaning and fulfillment. Work devotion stands in contrast to family devotion; historically the domain of mothers; family devotion is a cultural model that defines motherhood as a woman's primary vocation. From the late nineteenth century onward, it emerged from ideals of children as sacred (Zelizer, 1994), and media portrayals of breadwinning fathers and home-making mothers investing all of their emotional and physical resources into their children (de Laat & Baumann, 2016; Denny et al., 2014; Hays, 1996). Taken together, these devotion schemas serve as cultural frameworks for making sense of the world. They are

“objective in the sense of being shared, publicly available understandings. They shape social structure, the patterns and activities of groups and individuals in institutions, firms, and families. They are also subjective and partially internalized, thereby shaping personal aspirations, identities, and desires.” (Blair-Loy, 2003, p. 5).

Devotion schemas are thus understood as influences on internal, individual action based on external, shared culture. This perspective can be conceptualized as a culturalist approach, viewing schemas as “sets of taken-for-granted ideas that guide action “from the outside in”” (Leschziner & Brett, 2021, p. 1210).^2^

Recent research on work and family devotion shows that these schemas persist for both men (Blair-Loy & Williams, 2017b; Brumley, 2018) and women (Blair-Loy & Cech, 2017). Highly educated women who return to work after dedicating themselves to childcare still choose jobs and schedules that allow them to prioritize their families and maintain their privileged social class status (Stone & Lovejoy, 2021; see also Stone, 2007). Men in high-status positions conflate work devotion with productivity and competition, which diminishes their opportunities for involved fatherhood (Blair-Loy & Williams, 2017a).

Although work and family devotions continue to exert strong influences on work and family life, other research points to alternative parenting ideologies guiding career decisions (Christopher, 2012; Damaske, 2011; Gerson, 2009; Uttal, 1996). Dow (2016) argues that intensive mothering and family devotion are not monolithic influences on all parents; Black mothers have long repudiated dominant parenting ideologies in favor of ideas about good parenting that align with their cultural and community experiences (Collins, 1990). Other labor market changes likewise call into question the extent to which work and family devotion is experienced as competing. In Canada, as of 2015, 75 percent of dual-earner couples with children had two full-time working parents, and in the United States, the figure is closer to 50 percent (Pew Research, 2015; Statistics Canada, 2016). In each case, the majority of these partnerships are different-sex couples among whom the division of childcare and housework tends to be more contentious and unequal (Goldberg, 2013). While women who espouse family devotion may have previously worked part-time for extra income, they are now increasingly working full-time hours, alongside their family and household responsibilities (Guppy et al., 2019). In many countries, women's full-time employment is supported by work and family policies (Collins, 2019; Misra et al., 2011; Moen et al., 2013), and by increasingly egalitarian gender ideologies (Pfau-Effinger, 2012).

## Gender and Family Time

At the same time while men remain the prototypical ideal worker, fathers’ involvement with children is increasing (Ball & Daly, 2012; Maume, 2016; Raley et al., 2012; Ranson, 2012). The image of fathers as breadwinners and disciplinarians has given way to one of fathers as engaged caregivers who play with their children, comfort them, read to them, and change diapers (Milkie & Denny, 2014; Scheibling, 2020). These changing cultural perceptions are mirrored in shifting patterns of housework and childcare responsibilities among both women and men. While the balance of household labor and childcare remains tilted towards mothers, fathers are increasing their share of childcare, spending an average of three hours during the workweek on childcare compared to mothers’ 3.8 h (Kaufman, 2013; see also Guppy et al., 2019). The shift towards more involved fatherhood suggests that men may be less inclined to put in long hours at work. Research provides some evidence of this, with many men reporting a desire to spend more time at home (Damaske et al., 2014; Pollmann-Schult & Reynolds, 2017).

Despite the desire to be more engaged in childcare and housework, half of working parents, including fathers, report time shortfalls with their children (Milkie et al., 2019). This indicates that the evolving expectations around involved fathers and full-time working mothers may not align with the challenges that workers face in their daily lives (Brumley et al., 2021). Indeed, research suggests that when fathers attempt to be more involved at home, they encounter stigma and career setbacks (Chung, 2022; Rudman & Mescher, 2013; Vandello et al., 2013), while working mothers continue to be more likely to prioritize their caregiving responsibilities over paid work (Daly, 2002; Hilbrecht et al., 2008; Sullivan & Lewis, 2001).

Given the tensions between shifting labor market dynamics and changing parenting norms, understanding the cultural forces behind overwork takes on new relevance (Golden, 2009). This article investigates whether work devotion schemas persist in serving as a guidepost for those subject to working long hours. I found that while some employees work as hard as they do because of the work devotion they embrace, a sizable number espouse devotion to both the realms of work and family. These dual devotions are realized through the pursuit of work–life integration, which is in turn facilitated through flexible work arrangements. However, while dually devoted fathers engage in remote work to spend more time with their children, dually devoted mothers report doing so in order to accomplish more paid work. Dual devotions thus enable men and women to enact idealized dispositions towards family and work that defy societal norms about the gendered allocation of family- and work-time. In actuality, however, neither group scales back their paid work hours. These findings support the argument that flexible work arrangements, ostensibly designed to reconcile work/family conflict, facilitate work intensification (Chung, 2022; Glass & Noonan, 2016; Hilbrecht et al., 2008; Kelliher & Anderson, 2010; Kossek et al., 2010). They also reinforce the notion that overworking is a prerequisite for conforming to the ideal worker image (Reid, 2011, 2015). The culture of overwork is reproduced even by those we might expect to resist it—privileged and devoted parents—and this is manifested in gendered ways.

## Data and Methods

In 2018, I was invited by an executive to conduct interviews with IT employees at a multinational financial services company located in a large metropolitan area in Canada. The company was in the midst of an organizational and cultural transformation where the goal was to introduce more agility and flexibility; they were adopting workflow methodologies that originated in small Silicon Valley tech start-ups and moving away from office cubicles in favor of open concept workspaces without assigned seating (de Laat, 2023). The executive was part of a committee seeking to understand barriers to women's advancement. In Canada, as elsewhere, women confront discrimination in the field of IT, and structural barriers to career advancement persist (Alegria, 2019; Mickey, 2019, 2022; Wynn, 2020).^3^ In exchange for the opportunity to interview employees, I offered to write a report of my findings regarding barriers to women's advancement.

To recruit participants, the HR department sent an email to 200 IT workers with similar seniority levels, earning between $70,000 and $110,000 annually, and invited them to participate in an interview about flexible work arrangements and work–life balance. Because the participants are salaried, they do not earn overtime pay for putting in longer hours. This sampling criterion attenuates the influence of economic necessity on overwork. The recruitment list included variation across parental status and gender, and it prompted a response from 92 employees. Interviews took place with 41 women and 43 men at various office locations around the city (*N* = 56) and via telephone (*N* = 28) and lasted on average one hour. The interview guide included questions about what makes a good worker and a good parent, personal goals and career plans, perception and use of flexible work arrangements, work hours, approaches to parenting, and relationships with managers and co-workers. All but two interviews were recorded and transcribed.

After the completion of each interview, I wrote a research note summarizing the key points. The research notes were used to create a database that included codes for various attributes such as remote work and flextime use, division of household labor, career aspirations, work hours, gender, marital and parental status, race/ethnicity, age, job title, and organizational tenure. Some qualitative researchers contend that meaning saturation, whereby the meanings attributed by interviewees to certain phenomena become patterned, can be reached after conducting a range of 16–24 interviews (Hennink et al., 2017). Because the interview sample design included both men and women who were parents or child-free, I did not detect meaning saturation in my research notes until after approximately 70 interviews had been completed. I continued to complete all scheduled interviews, for a total of eighty-four. Meaning saturation was met through the identification of similar responses to questions about flexible working practices (regarding either their necessity or disdain for them) and what makes a good parent (regarding how norms have changed and the importance of time).

Data analysis followed the methods outlined by Deterding and Waters (2021). The initial coding phase involved index coding, which categorized extensive text portions based on interview guide questions and recurring themes identified during research note writing. For example, while writing the research notes, I noticed variation in expressions of working to live or living to work, and statements concerning flexible work policy usage. Index coding thus included the latter themes, among others, as well as tracking the number of hours worked and other demographic information.

The subsequent stage of coding was more focused and involved a deeper analysis of analytically interesting index codes through the development of sub-themes (Emerson et al., 1995). This included an analysis of participants’ motivations for working remotely and reasons for living to work, revealing themes of work and family devotion, work–life integration, and the enabling potential of flexible work arrangements. A third and final stage of coding entailed refining and validating the stage two coding by distinguishing patterns across groups based on attributes such as parental status, gender, and work hours.

Table 1 outlines demographic details of the interviewees. I requested to speak to people of all genders, though all participants identified as cisgender. Fifty percent of participants identified as a person of colour, which is in line with the broader demographics of the city where the company is located. Research on the work/family interface documents a relationship between race and dispositions towards work and family obligations (Dean et al., 2013; Dow, 2016). By contrast, the third iteration of coding in the present study did not reveal any variation in espousals of work and family devotion or preference for work–life integration by race/ethnicity (see also Mosseri, 2021). The more salient variation, and thus what the following analysis examines, is gender.

**Table 1.** Descriptive Overview of Interview Participants.

	Raw counts	Percentage of the sample
Women	41	49
Men	43	51
People of color	42	50
White people	42	50
Parents	65	77
Non-parents	19	23
Age (years)		
20s	5	6
30s	21	25
40s	30	36
50s	21	25
60+	7	8

## Findings

### Working to Live

Interviews with IT employees revealed two broad patterns in dispositions towards work: those who prefer to work to live and those who live to work. As indicated in Table 2, the latter are subject to overwork, averaging 50 h of paid work per week. By contrast, those preferring to work to live reported working 40 h per week. They were older on average, had a longer work tenure, and some were close to retirement. They were looking forward to receiving their pension, and because they had no desire to progress within the company, they scaled back their work responsibilities. Others had suffered health setbacks, which motivated them to prioritize their mental and physical health by resisting overwork. When asked about where they envision themselves five years from now, a response like Jeanette's was common: “I want to be retired [laughs]. I have no desire to move up the ranks. I’m quite happy where I am.” In responding this way, Jeanette associates her goal of retirement with her desire to stay in her current role as an analyst.

**Table 2.** Variation in Self-Reported Work Hours and Division of Household Labor.

Disposition toward work	Average reported weekly work hours	Reported division of housework and between respondent and spouse
**Work to live**		Childcare was either not applicable for this group (some of whom were older) or was shared evenly. Most reported an even division of labor. Those with spouses who were already retired reported doing less housework. No gender variation in responses was identified.
Men (*N* = 9)	40	
Women (*N* = 17)	40	
*Family devotion*		Traditional division of labor; though they work full-time, each woman reported responsibility for most childcare and housework.
Women (*N* = 3)	39	
**Live to work**		
*Work devotion*		Men reported a traditional division of labor, with their spouse taking on the majority of housework and childcare, or having it outsourced to cleaners and nannies. Women reported an equal division of labor, supplemented with outside help.
Men (*N* = 17)	51	
Women (*N* = 6)	55	
*Dual devotion*		Both men and women reported an equal division of labor, and some report supplementing this with outside help.
Men (*N* = 17)	50	
Women (*N* = 15)	49	

A final characteristic shared by most of those working to live was a lack of family obligations. Family was mostly mentioned in passing or when prompted, but was not otherwise something that factored into respondents’ day-to-day concerns. This may be a result of their life-course location; most had adult children, and parenting was no longer a daily activity to be kept top of mind.

In contrast to the latter, a small subset of those working to live—three participants, all mothers—expressed a singular devotion to family. Each worked part-time while their children were young and had recently started working full-time. Diane, an IT risk manager, transitioned to full-time employment after years of working part-time when her children were young in order to increase her retirement savings. Even though she now works full-time, she and her husband maintain an uneven division of childcare and household labor: “when it comes to the household, my husband will do groceries and cook some meals. But other than that, it's mom. And I’m okay with that. I’m okay to be the home base, you know.” Diane shared that even though she worked part-time when her kids were young, she feels guilty for not being a stay-at-home mom: “If finances weren’t an issue, I’d be gone tomorrow. But the fact that we’re two incomes with very busy kids, and we’re not too far off from college, I gotta stay.” Lenore spent her career job-sharing, working two days one week and three days the next. She was offered a job that could only be completed full-time and accepted it reluctantly. When asked about her decision to job-share, Lenore replied:

Honestly, I don’t know how people with kids and full-time jobs do it, because you never see your kids. In a demanding job, you just never see your kids. I didn’t want that to happen to me, so I was willing to make some adjustments in order to be able to spend more time with the kids and be able to go in and volunteer at school and go on trips.

Diane and Lenore cannot fathom being committed to both work and family. They would prefer to dedicate more time to family life; however, their financial obligations prevent them from doing so.

In summary, those preferring to work to live are able to maintain standard work hours and avoid overwork. The presence of this subgroup suggests that overwork is in part something that employees select, and in the remaining analysis, I focused on why and how certain employees accomplish overwork.

### Living to Work: Work Devotion

Like those expressing a singular devotion to family, men and women espousing work devotion also perceive work and home as too time-intensive and emotionally absorbing to enable adequate commitment to both simultaneously. Though they may have children, those devoted solely to work allow others to provide childcare and work an average minimum of 50 h per week. This group of participants did not believe it was possible to be career-focused and family-focused at the same time. One extreme example of being devoted solely to work is Sandra, a self-professed workaholic, mother of two adult children, and director with over one hundred staff reporting to her. Sandra reports working 16 h a day, seven days per week. Regarding her approach to working long hours, she said:

I come from the old school, where the mentality is you have responsibility and you do everything in your power to nurture that responsibility. My dad was not a man who tolerated softness and I’ve been told many times I’m tough. But that's just who I am, right? My staff will say to me, “You’re still here at 7 o’clock?’ Yeah, I need to know that the work is going to get done, and I want to be here to direct should I need to make decisions. They may say “Oh Sandra, but your days are so long.’ Yeah, I know. It's okay because I don’t take my responsibilities lightly and I make sure I set an example for the staff so they understand. We’re dealing with a lot of money so I think you lead by example. You can’t have this “do as I say, but not as I do’ mentality. No. I want you to do like I do and I want you to do as I say.

The fact that Sandra's children are grown enables her to work long days. However, even those with young children espouse a singular devotion to work. Some harbor biases towards co-workers who take measures to accommodate their family lives. Dave, a director, has two school-aged children and a five-year plan to become a vice president. His wife works part-time, which, he acknowledges, enables him to work longer hours. When asked about his approach to working, he shared:

At a certain time in your career you have to make a choice between doing all the family stuff or doing all the work stuff …You have to decide whether it's, you know, giving up something to move ahead. And I would never have anyone actually admit to that. To say you know your career is being held back ’cause you’ve got kids, but I – I get that impression sometimes that I’m competing against someone who doesn’t have kids who can work twenty-four hours a day if they wanted to.

Dave is motivated to work long hours partly out of fear that he is competing against employees without family responsibilities. As someone with a work devotion schema, he also believes it is impossible to be fully engaged as a parent and to give 100 percent to his job. Such singular devotion to work is fueled by the mental model of the ideal worker who puts in long hours at the office, and it is upheld through the often-invisible support that his wife provides behind the scenes.

### Living to Work: Dual Devotion

In contrast to those espousing devotion to work or family, a subset of interviewees did believe it was possible to fully devote oneself to a career and family life. This is not a new revelation, as Blair-Loy (2003) documented in her research over twenty years ago a small group of mavericks who challenged the cultural ideals undergirding devotions to work and family by combining part-time paid work with family life. What differentiates the employees espousing dual devotion is the scale at which they experience the combination of career and family; not only do they work full-time rather than part-time, but they work more hours than they are contractually obligated to. Not only do they pursue parenthood, but they also engage in intensive-parenting through significant financial and emotional investment in their children. They strive for perfection in both work and family life (Beckman & Mazmanian, 2020). In the following sections, I document the mechanisms that facilitate dual devotion, and consequently, serve to reproduce overwork.

Thirty-two interviewees espoused a strong devotion to both work and family. As ideal-typical schemas, devotions to work and family vary in practice. In Table 3, I conceptualize these devotions as located across a spectrum, with singular devotion to work and family at either end, and dual devotion occupying a middle space, where certain traits associated with either work or family may be more or less salient.

**Table 3.** Characteristics of Work/Family Devotion Schemas.

	Family devotion (*N* = 3)	Dual devotion (*N* = 32)	Work devotion (*N* = 23)
Characteristics	Childcare and household responsibilities are a calling, requiring single-minded focus and allegiance. It is enabled by having a spouse devoted primarily to work (Blair-Loy, 2003).	Family and work are viewed as simultaneously fulfilling and meaningful and are pursued with similar levels of passion. Participants work long hours and express a desire to move up the ranks. They also report going to great lengths to be available for their children and support them in extracurricular activities. Parenting ideals are achieved through concerted cultivation (Lareau, 2003). Dual devotion is accomplished though work–life integration, which is in turn facilitated through flexible work arrangements.	Career is a calling requiring single-minded focus and allegiance. It is enabled by having a spouse devoted primarily to family, and/or by outsourcing housework and childcare (Blair-Loy, 2003).
Example	“Mom is the nurturer in our house, you know … I took mat leave with both kids. And the kids were 18 months apart so I pretty much didn’t want to work after that. I was like, can I just be a full-time mom?” *Diane, IT risk manager*	“One of the reasons we decided we were only going to have one child is because I wanted to have a career and I wanted to be able to work. I wanted to be able to give my all to my kid, but also my job.” *Deepa, senior manager*	“I really love the work and look forward to progressing within the organization. I absolutely, in many cases, prioritize work over family life.” *Matthew, senior director*

Devotion to work was demonstrated by bringing work home, texting bosses throughout evenings and weekends, and working an average of 50 h per week, well over the mandated 37.5 h. Many spoke passionately about their own managerial style and work philosophy, happy to be speaking to someone eager to hear about their approach to work. Participants also reported a desire to move up the ranks, envisioning themselves as directors or executives.

One such example is Adam, a director and father of two young children, who reports working 50 h per week. Prior to his current role, he was the only one on his team with a specialized skillset concerning security operations. He worked such long hours that he finally had to tell his manager that he could only continue if he worked from home. For one year, he worked 12-h days. Working those long hours was worth it because he was subsequently able to justify his need for a team and built a unit of staff dedicated to his projects. Employees like Adam, however, were equally happy to speak with someone eager to hear about their family life. They delight in organizing their children's extra-curricular activities, mapping out family activities on the weekend, express thoughtful parenting philosophies, and go to great lengths to ensure they have the right childcare in place. When I asked Adam what makes a good father, he reflected:

Both of my parents worked outside the home, and so being there for my kids is very important, and kind of working with them through emotional difficulties. We’re trying to explain feelings to them because emotional intelligence is a very hard thing to grasp and that's one of the things I want to make sure they try and learn about. It wasn’t something my parents dealt with and I can’t think of many parents that did that then to be honest.

Like others espousing dual devotion, Adam has spent time reflecting on his own childhood and uses that as a measure for his own parenting philosophy. Contrary to traditional fathers in different-sex partnerships who may coach sports teams and engage in public fatherhood but leave primary care responsibilities to their spouse (Kaufman, 2013; Kelly & Moen, 2020; Shows & Gerstel, 2009), Adam willingly takes on the emotional responsibility of nurturing (Doucet, 2006).

Despite working full-time, interviewees embody many of the traits that are associated with intensive mothering; their approach to child-rearing is “child-centered, expert guided, emotionally-absorbing, labor-intensive, and financially expensive.” (Hays, 1996, p. 8). While the latter traits are associated with motherhood, male participants embody the same intensive parenting ideologies (see also Guppy et al., 2019). Ahmed, a director and father of three, described the many activities his children are enrolled in:

My son plays travel basketball, so that's a big commitment, and the girls have their stuff. My middle daughter's got track. All three kids have math academy. And then the oldest coaches soccer, the middle one babysits, so it's lots of movement … I would consider our scramble to be a little bit luxurious. There's stuff we could take off of our plates, but we choose not to, whereas I think in my parents’ generation had no choice. There was nothing they could actually take off their plate. So, we sort of take it as a blessing.

Ahmed's family devotion was evident through the expression of concerted cultivation, a parenting style that fosters children's talents by incorporating organized activities into their lives (Lareau, 2003). In line with the social class location of the interviewees, this is a middle- and upper-class practice that is also characterized by consciously developing language use and the ability to interact with social institutions. Parents like Ahmed reflect amicably on the frantic pace that their kids’ activities introduce to their lives and even welcome it as a privilege.

### What Facilitates the Dual Devotion Schema?

Parents with dual devotion schemas share in common preference for integrating work and life and perceive these two spheres as intertwined. This integration is motivated by a desire to resolve some of the conflicts resulting from holding multiple roles—for example, being a “father’ and a “project manager’ (Rothbard et al., 2005; Williams et al., 2016). It can involve adopting the same emotional or intellectual approach to handling either realm (Nippert-Eng, 1996), as when managers describing their method for handling direct reports at the office as similar to parenting. Natasha, a senior manager and mother to an adult son, believes that being a mother positively impacts her ability to manage effectively:

If you think of parenting as being about convincing your kid to do the right thing, then when you hear a kid who says, “You can’t make me. You are not the boss of me,’ you have to learn techniques and strategies for getting them to do what you want, but because they want to, not because they feel like they have to. It's the same thing as a manager. For me, that way of influencing someone, understanding where they’re coming from, thinking, “Okay, I'm pretty sure he's not going to want to do that. I better think in advance of how I'm going to convince him."

Natasha indicates her willingness to integrate her parenting and managerial philosophies by applying the same tactics of persuasion on her reports as she does on her son, in order to motivate them.

Among those espousing dual devotion, integration is facilitated through the use of flexible work arrangements: working remotely, and choosing when to start and stop work. Participants describe weaving work and family responsibilities: leaving work at 3 pm in order to drive their children to extracurricular activities, and continuing to work from home into the evening. Ahmed's example represents a typical way to integrating work and family life:

I had to take off at three o'clock because my son had basketball. And I have a document that I have to share at three o'clock with a few of my sponsors. So I had no choice, last night I had to pull out the laptop, and I worked on it for four hours. And I probably still have about another 20 min I just want to glaze over a few things on it after this call. And so, you know, I will be skipping out earlier than I normally do. But I still put four hours in last night and I will work some more tonight … it's a trade-off, right? The company is reciprocating flexibility to me, and I have a goal that I need to achieve, you know, so do I gotta do that as well.

As work and family scholars have demonstrated, flexibility is perceived by workers like Ahmed as a gift; in return for its continued use, he consents to work intensification and longer hours (Chung, 2022; Kelliher & Anderson, 2010).

Equally common is fully integrating the realms of work and family together by working from home anywhere from one to three days per week, on average. Participants report working from their kitchen tables and taking time to throw in a load of laundry while on conference calls. Parvati is a senior manager who regularly works 50 h per week. She tries to be very involved in her son's life, ensuring to always be the one to pick him up and drop him off at school, even though the school is close enough for her son to walk. She makes sure to put money aside so that they can travel together every March break. In terms of her career, Parvati would like to move up the ranks and thinks that as long as her work ethic remains visible, she can achieve this:

“I have a strong work ethic. Even when I vacationed this week! I was on vacation in New York City with my son and I called in to a meeting for five hours on Saturday morning. And Thursday, there was an issue, an email going back and forth with something, and I was responding to the email. I was on a call with my VP on Thursday. I was on a call Friday with my manager. So it's like I feel like I’m accountable for this and I need to make sure it's done properly.”

Parvati works remotely two days a week, reporting that “Working at home is better because you have a bit more balance, like you can fold the laundry or cook a meal in between phone calls or whatever you’re doing.” Parvati exemplifies the autonomy paradox*,* where work–life balance is less about scaling back paid hours of work and more about having the autonomy to fulfill household tasks and responsibilities over the course of the workday (Chung, 2022; Lott & Chung, 2016; Mazmanian et al., 2013).

While both dually devoted mothers and fathers espoused a preference for work–life integration and used flexible work arrangements accordingly, they did so for different reasons: Fathers reported engaging in flextime and remote work in order to spend more time with their children, whereas mothers reported doing so in order to squeeze in more hours of paid work.

*Fathers’ expression of dual devotion.* Fathers with dual devotion possess many of the characteristics associated with new norms of fatherhood; those with babies and toddlers describe sharing night shifts with their partners to feed and comfort the baby, many of them took parental leave after the birth of their children, they report sharing housework evenly, and they share in the work of shuttling their children to school and extra-curricular activities.

Brad, a 38-year-old project manager, is actively strategizing his career progression. He recently took on a new position in order to learn about a new application that the company heavily invests in, thinking that “it would be exciting to get in on the ground floor.” His plan is to stay in this role for one year, and then apply for progressively more senior positions in management. At the same time, Brad is invested in his career progression, and he is heavily invested in family life. He reports that his division of housework is shared evenly: he does all of the cooking, meal planning, and grocery shopping. His wife does laundry, and they split doing the dishes and dusting. A cleaning service every two weeks takes care of the rest. Growing up, Brad's father wasn’t around much, and it wasn’t until he became a father himself that he realized he missed having his dad around. Therefore, he tries to be present for his daughters. He attends one field trip for each daughter every year, and he is part of a club for fathers called “Dad's Club of Morrisville,” for which he does fundraising and organizes playdates. At the time of our interview, Brad was working remotely on Thursdays but was in the process of switching it: “I usually work from home on Thursdays, but I need to switch it to Tuesday because of after-school activities that the kids have. That day, they both start at five o'clock. So, one of us needs to be at karate, and one of us needs to be at piano.” Working remotely thus enables Brad to accommodate his children's extracurricular activities and simultaneously maintain focus on his career.

Dually devoted fathers often mention starting work early, squeezing in emails, and planning their day at home before their children wake up, pausing work to get their children ready for daycare or school, and then picking up work again. It can also mean leaving the office at 3 p.m. in order to pick up children after school and working in the evening again once the kids are asleep. Regarding this interweaving of work and family responsibilities, Kevin, a senior manager and father of three children under the age of eight, related the following:

I started getting up at 4 a.m. I actually will get up and I'll work for a bit. When you get up in the morning, even if it's stuff from the day before, you're getting a head of the day. And so, a long time ago when I was in grad school, I had a job as a handler for a billionaire. And this guy got up every morning at 2:30 a.m., and he’d work out, and then he’d start work at 3:30/4:00 a.m. And I said to him one day, I was like, “Why are you doing this?” and he said, “If you want to beat other people, you gotta get up before they do. So I built my empire being up before my competitors.” And so, I finally have enough energy to do the same thing. I'm not trying to beat anybody, I'm just trying to get ahead of the day, and it actually works … the stuff that might take you a long time, seems like a drag, you kind of look at your day like, “oh, these are the things planned.” And you come into work, you're already executing on that stuff, because you've already thought about it.

Kevin reports that he does the grocery shopping, and he and his wife take turns cooking. He is responsible for getting his kids to school and daycare in the morning, and he stops work every day at 5 p.m. to spend evenings with his children. Spending evenings with his children is tremendously important to Kevin, and when asked what makes a good father, he shared:

You gotta be there. Here's a great one for you: How does a child spell love? And that's the number one thing. Right? I got that on a website, and it's called Supercharged Dad and I try to read that stuff. And it's – I read that in there, and I was like, “Shit. That's so true. Time.”

For Kevin, having autonomy over his work hours––working in the morning while his children sleep––enables him to maximize time with his children, which is one of his core values as a father. This perception, however, stands in tension with the fact that such autonomy leads to overwork and self-exploitation (Chung, 2022; Mazmanian et al., 2013).

Thomas, a director, similarly prefers to integrate work and family responsibilities. He works from home once or twice a week, and on the days he is in the office, he leaves work at 3 p.m. to pick up his kids from school and works throughout the evening. When asked what the ideal work–life balance looks like, he said:

Work-life balance is more of a having the best living choice I think rather than having something that is rigid … I think the flexibility of choice would define work-life balance for me … I am putting long hours in, but it's more like we got a block here, and then an hour off and then this block here. And that tends to work.

For Thomas, Kevin, and the dually devoted fathers who use flexible work arrangements, interweaving work and family responsibilities enables them to feel as though they can do their best as employees and spend time with their children.

*Mothers’ expression of dual devotion.* Dually devoted mothers also reported a preference for work–life integration and used both flextime and remote work policies at similar rates to their counterparts. The similarities between dually devoted mothers and fathers diverges, however, when we examine their reasons for working at home; most dually devoted mothers report choosing to work remotely in order to accomplish more paid work. This phenomenon aligns with survey data indicating that flexible work arrangements enable mothers to remain financially competitive in the labor market (Chung & Van der Horst, 2018; Fuller & Hirsh, 2019; Van der Lippe et al., 2019).

When asked how she likes working from home, Marlene, a business analyst and mother of two school-aged children, shared:

You get more done. You don’t mind working a couple of extra hours. I can get on the computer at 8:00 or 8:30 instead of sitting in traffic. And I don’t mind working until 6:00. I can go make dinner and I don’t feel guilty about doing that and then going back down for a couple of hours to finish up whatever is required for the day.

Such emphasis on productivity instead of childcare or child-centric activities was common among dually devoted mothers. Marlene acknowledges that the flexibility enables her to provide indirect care through food provision. However, whereas dually devoted fathers express a preference for flexible work arrangements because it helps them overcome guilt about not being involved in direct childcare, for Marlene and other dually devoted mothers, remote work helps them overcome feelings of guilt about not dedicating enough time to their paid work. Like most guilt, Marlene's is not rooted in objective truth, as she averages 48 h per week, and occasionally, more: “I had two weeks where I was working 55 h. The work has to get done. There are deadlines. There are expectations. You can’t just not do it.”

Raya, a project manager and mother to a seven-year-old daughter, remarked how different parenting is today compared to when she was younger:

Growing up, my mom would pick us up from the school and just provide food. She made sure we were doing our homework. Whereas my daughter expects certain things to be done but food on the table is not one of them. For her, mom is somebody who is providing, who is taking her places.

Raya's daughter is enrolled in swimming, art, and dance classes, and more recently, a tutoring program. She used to pay someone to clean her house biweekly but has since reallocated that money towards the tutoring lessons. Raya's investment in her daughter is emblematic of concerted cultivation, owing to her willingness to make sacrifices in order to invest heavily in expert guidance to enrich her daughter (Hays, 1996; Lareau, 2003). She averages a 50-h workweek, which includes working occasionally in the evenings, and several hours each weekend. She makes use of flextime, starting and stopping work as needed, and works remotely. It is the latter that enables Raya to put in the hours she feels are necessary to lead to a promotion from a junior to a senior project manager:

Where I need the flexibility, I am getting it. Being at the level I am, having the responsibilities I have, it is stressful but overall, I think I already have that flexibility. When I am [working from] home, I can work until 6:00 or 7:00. I am working more when I am working from home. That kind of flexibility is what I need in my situation, and I have that. Everybody, whether it is my HR, whether it is my director, everybody is supportive and helpful in that way.

As with all the employees I spoke to, Raya is on salary, so her longer hours do not earn her more money. Nevertheless, she conveys gratitude for the flexibility that allows her to put in longer hours (Kelliher & Anderson, 2010).

Tanya is an analyst, a mother of four school-aged children of whom she has sole custody, and a stepmother of three children. In addition to her regular workload, she is currently enrolled in a coaching academy to gain certification in specialized managerial work. It is a new program that had over 500 applicants, and she was one of only 15 selected. She manages to squeeze in some of her course reading in between meetings but completes most of it at night. She also oversees her children's extracurricular activities which include karate, hockey, soccer, and music classes. She works from home twice a week and occasionally after her kids go to bed. Of her work hours, she shared:

I work in the evening once the twins are in bed, probably only twice a week. I’ll log in if there's a deck that I need to finish or something. On the days that I work at home, I log in at 7:30 in the morning and I usually don't log off until 5:00 because I don't have the commute, right? So that's good.

As with many people who work from home, Tanya reports working longer hours on the days she does not commute to the office, despite her busy home life (Chung, 2022; Mehdi & Morisette, 2021). While she appreciates the ability to work from home because it makes things easier, her focus throughout the interview was on how remote work enables her to complete more work and less on how it enables her to engage with her children:

I'm always, always busy. Right now, it's my own fault, obviously because when I'm working at home in the summer, the kids are home. They see me home, but I'm working, so I can't do anything with them so it's kind of like this, “Mom's here, but she's not doing anything with me,” right? That's not their fault. It's not my fault. I work and I've also tried to explain to them, and one day they will get it. I, like most parents, work.

Although working from home means that her children do not have to receive childcare outside of the home during the summer months, the time Tanya saves by not commuting to the office is allocated to the completion of more paid work.

## Discussion

**Figure 1.**
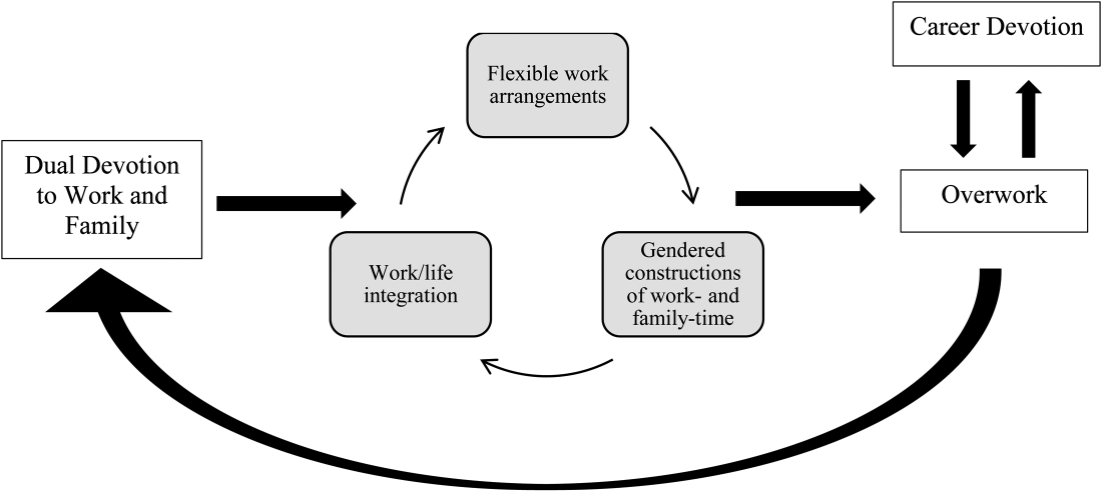
Process model of the relationship between dual devotion and overwork.

Figure 1 outlines the process through which overwork is reinforced. Among those who profess living to work, many perceive their career as a vocation that requires singular focus. Career devotion remains a powerful schema that directly guides commitment to overwork, and this relationship is bi-directional. Others who claim to live to work choose vocational commitment in two domains, work and family, and submit to both wholeheartedly. As Alice, a director, put it succinctly, “For me, I wanted children, and I love my career. I want my career too, so we’ll make it all work.” The pursuit of “making it all work” remains feasible due to the self-reinforcing relationship between a disposition towards work–life integration, which includes overlapping boundaries between one's role as an IT professional and one's role as a parent; the use of flexible work arrangements to fulfill both family and work time demands; and gendered constructions of how one is committed to work and family that defy common stereotypes about the roles of mothers and fathers in society. Taken together, these cultural dispositions and workplace policies facilitate overwork, and in turn, reinforce dual devotion both to work and to family.

Notably, the dually devoted women and men I interviewed report working a similar number of hours, averaging 49 and 50 h per week, respectively. Men report that flexible work enables them to spend more time with their children, and women report that flexible work enables them to spend more time working; however, neither group is scaling back their commitment to the other realm. Rather, both men and women espousing dual devotion perceive work–life integration and remote work as the means for enabling idealized relationships with family time and work time. This allows them to enact identities that wider society has been slow to acknowledge: involved fatherhood for men, and career ambition for mothers.

Because women have traditionally been responsible for provisioning childcare and housework, their contributions to that realm are taken for granted. Flexible work is not conveyed as something that facilitates time for childcare because for working mothers, childcare remains a non-negotiable duty, something that most others expect them to do, and that they expect themselves to do. In addition, as in other fields of work, women in IT have to work harder in order to be deemed competent, and putting in long hours is one way of demonstrating commitment (Cooper, 2000). Dually devoted women thus have added pressure towards overwork, and the narrative of remote work as facilitating longer hours may be a strategy for conveying competency and commitment.

Similarly, men remain the prototypical ideal worker. For the dually devoted fathers I interviewed, flexible work is not conveyed as a means of facilitating more time for work since putting in longer hours is expected of them. Because norms of fatherhood are changing and men both want to be more involved at home and are expected to be, their reports of work–life conflict are increasing (Aumann et al., 2011). Internalizing the narrative that remote work and flextime enable more time at home similarly fulfills dually devoted fathers’ desires to feel and be perceived as caring and involved parents. Dual devotion schemas, flexible work arrangements, and the social constructions of time through which they are enacted assist working parents in achieving idealized relationships work and family time, albeit in ways that vary by gender. The enactment of dual devotions also suggests that working parents are spread thin, and this may have consequences for their health and well-being.

## Conclusion

Conceptually and empirically, this study identifies dual devotion as guiding commitment to work and family and makes three contributions to scholarship on work and family inequalities. First, dual devotion reveals how cultures of overwork are also reproduced by those with responsibilities and desires to be involved in family life. While the ideal worker has long been identified as a prototype that fuels unreasonable and unrealistic expectations for all workers, this article demonstrates how ideal working parents—those espousing dual devotion—may also contribute to unrealistic expectations. Because they find the pursuit of dual devotion self-fulfilling (or at the least, necessary), there is less likely to be opposition to overwork from this population. Moreover, because those espousing dual devotion are ambitious and have managerial decision-making authority, their actions are more visible and powerful. Even though those preferring to work to live (rather than live to work) may not share a disposition towards overwork, but they are nevertheless subject to their superiors’ and coworkers’ overwork as the baseline against which their behavior may be compared. This is the case irrespective of gender. Given that no one is actually reducing overwork in an effort to accommodate unpaid care obligations, employers continue to benefit (Chung, 2022; Mazmanian et al., 2013).

Adherence to dual devotion schemas serves to reproduce workplace inequality and overwork by facilitating the feeling—real or perceived—of devoted parents being able to “do it all.” “Doing it all” has implications for social class reproduction, as the relationship between overwork and the active pursuit of family time is self-reinforcing: experiential dimensions of time are dependent on financial resources to pay for family activities and outsourcing housework and childcare (Hays, 1996; Jacobs & Gerson, 2004), which may in turn reinforce the drive to invest in one's career. There are only so many hours in the day, and those wishing to be engaged at home invest effort in managing the effective use of home time. For dual-earning parents, scheduling family time can be a priority imbued with financial, social, and cultural capital, where busy family schedules packed with activities become symbolic of success (Daly, 2002; Kremer-Sadlik et al., 2008; Kremer-Sadlik & Paugh, 2007). Consequently, managing family time increasingly includes project management tactics, such that many North American families experience a “Taylorization of the home” (Daly, 2002, p. 339), where “triaging” between competing priorities is normalized (Carrigan & Duberley, 2013). The positive valence attributed to scheduling activities that are culturally enriching, yet inaccessible to low-income families, may serve to reinforce social class inequalities (Lareau, 2003; Tubbs et al., 2005).

Second, this study extends our understanding of family-based Taylorization by explaining how gendered perceptions of time are constitutive of the dual devotion schema; dually devoted fathers perceive themselves to be equally involved in home life, through organizing activities, reading parenting blogs and books, and spending time engaged in childcare. This finding aligns with broader trends toward norms of involved fatherhood (Kaufman, 2013), particularly in Canada (Ball & Daly, 2012; Guppy et al., 2019; Shafer et al., 2020). It also substantiates survey data showing that during the Covid-19 pandemic, fathers increased their involvement in childcare (Carlson et al., 2021; Chung et al., 2021; de Laat et al., in press; Shafer et al., 2020).

To be sure, perceptions of time use are just that: perceptions. A substantial body of research demonstrates that men contribute less at home compared to women when working remotely. For example, Sullivan and Lewis’ (2001) qualitative study of remote workers finds that women more often integrated childcare and housework responsibilities with their paid work throughout the day, whereas men maintained “industrial time” and managed to avoid increasing their involvement in unpaid labor. Similar results are identified in longitudinal (Chung & Booker, 2023; Kim, 2020) and cross-national survey data (Kurowska, 2020).

In addition, time dedicated to household and childcare labor is not always commensurable with the clock-time that dictates paid working hours (Brumley et al., 2021). Other conceptualizations of time may be bound up in devotion schemas, such as “process time,” an understanding of time that is “enmeshed in social relations” (Davies, 1994, p. 280). The Taylorization of family time stands in tension with childcare practices; as Doucet argues (2022, p. 3), “responsibilities for care do not unfold in fixed, linear time units; they are messier, more circular, relational processes that involve flow, constant movement, and temporal modalities (past/present/future).”

Regarding possibilities for increasing gender equality in care work, a best-case scenario is that dually devoted fathers’ perceptions of spending more time with their children means that, in lieu of industrial time, they are taking on these more cognitive temporalities of care that have traditionally been the purview of mothers (Daminger, 2019; Doucet, 2001). In a worst-case scenario, remote work fuels the illusion of more involvement at home, strengthening the resonance of dual devotion schemas and reproducing an unequal division of household and childcare labor in the process (Gerstel & Clawson, 2018; Offer & Schneider, 2011).

Finally, because those espousing dual devotion consent to overwork by embracing work–life integration and the flexible work arrangements that enable it, the present analysis supports research on the paradoxical role that flexible work arrangements play in workers’ lives (Chung, 2022). Flexible work arrangements, and remote work in particular, continue to be highly valued by employees (de Laat, 2023; Kelly & Moen, 2020). Under the right conditions, flexible work arrangements can be beneficial to employees (Michel et al., 2011, Perlow & Kelly, 2014) and to women in particular (Chung & van der Horst, 2018; Fuller & Hirsh, 2019). However, evidence suggests that for the kinds of employees studied presently (white-collar, predominantly senior managers) flexible work arrangements can exacerbate both stress (Glavin & Schieman, 2012; Moen et al., 2013; Schieman & Young, 2010) and gender inequality (Chung, 2022; Conzon, 2023; Hilbrecht et al., 2008; Sullivan & Lewis, 2001). My findings complement research on the flexibility paradox by providing empirical data on the Canadian context. The espousal of dual devotion indicates that, despite the potential for a more equitable division of housework and childcare wrought by remote-working fathers’ (self-reported) higher levels of involvement, flexibility persists in fulfilling employers’ needs instead.

The interplay between gendered perceptions of time, remote work, and overwork holds policy implications. Though most participants in the current study did not exhibit high levels of stress, existing scholarship shows that there is a negative association between role blurring and well-being (Schieman & Glavin, 2008, 2016). Without the ability to afford childcare and housework support, those enacting dual devotions may be vulnerable to stress, and such effects may be felt unevenly. For example,  Fan and Moen (2022) found that Black women transitioning to remote work during the Covid-19 pandemic reported working longer hours, on top of their unpaid care obligations.

There is nothing inherent about remote work or flexible work arrangements more broadly that causes stress. Rather, the stigma and long working hours that often accompany remote work exacerbate inequality along lines of race, gender, and social class (Gerstel & Clawson, 2018; Thébaud & Pedulla, 2022). Policies that serve to reconcile work and family obligations and challenge the ideal worker norm are critical for improving well-being outcomes (Chung, 2022; Gumy et al., 2022). In addition to the importance of paid parental leave for fathers and universal childcare (Doucet, 2021; Friendly & Prentice, 2016), policies that place limits on employer overreach may help alleviate the negative repercussions associated with flexible work (Kelly & Moen, 2020; Ravanera et al., 2022). For example, in recent years, the governments of Canada's most populous provinces, Ontario and Quebec, introduced “Right to Disconnect” legislation which mandates that employees do not engage in work-related communication and are free from the performance of work outside of their paid working hours.

As a qualitative analysis of one workplace, this study has scope limitations. First, it centers the experiences of mid- and upper-level management. These individuals have achieved some degree of career success; however, they are not yet at the executive level, which is the level of employment studied in much of the research on work devotion to date (Blair-Loy, 2003; Blair-Loy & Williams, 2017a, 2017b). It is possible that the ability to enact dual devotion stalls out once employees reach executive status. There is analytical purchase to be gained from employing a longitudinal perspective on dual devotion, charting how it shifts across career progressions and family trajectories. Second, my analysis centers on those who select into overwork. As Table 2 indicates, numerous respondents prefer to work to live, and manage to not engage in overwork. Many of these respondents were close to retirement age or had a spouse whose career they prioritized, which goes a long way in explaining their preference for working to live. Nevertheless, future research would be served by a comparative analysis of these two work–life preferences to glean further insight into how the social organization of work may influence such preferences, age and tenure notwithstanding. Finally, the choice to interview employees in a similar salary band with similar levels of seniority precludes analyzing variation between socio-economic statuses and work-family dispositions. Intersectional analyses that prioritize a variety of social class locations alongside race and gender may further improve our understanding of work and family devotions.

Research acknowledges that for women, work-family conflict is not the source of their stalled advancement at work. Rather, the narrative that work-family conflict is inevitable masks the true source of women's stalled advancement: a 24/7 work culture that places unrealistic expectations on workers, and women in particular (Padavic et al., 2020). Flexible work arrangements like remote work fuel the “hegemonic narrative” of work–family conflict as the source of gender inequality at work by providing the sense that balance is possible without scaling back overwork. This study demonstrates that even as fathers become more involved at home (or perceive themselves to be more involved), cultures of overwork may not be disrupted.

## Notes

1. Overwork is defined by some as working unsustainably and beyond one’s own capacity in ways that negatively impact physical and mental health (Golden, 2006, p. 62). This definition has similarities to the concept of overload, the feeling of having too much work to do, and too little time to accomplish it (Kelly & Moen, 2020, p. 4). The definition of overwork used in the present article refers to working more than 50 hours per week.

2. I agree with recent characterizations that culturalist conceptualizations of schemas more accurately capture “external traces of potential schemas, not schemas themselves.” (Leschziner & Brett, 2021, p. 1221). With that caveat in mind, I use the term schema and/or devotion as a stand-in for ‘external trace of a potential schema’ for clarity and brevity.

3. It is important to note that Canada’s social welfare system offers some protections against the overwork culture, including parental leave benefits with a “use it or lose it” provision for the second parent (typically the father in different-sex partnerships). Additionally, employees in federally regulated industries, including those in the present study, have the legal right to request flexible work arrangements, though managers maintain the right to refuse such requests.
